# Self-help therapy for sleep problems in hospital nurses in Japan: a controlled pilot study

**DOI:** 10.1007/s41105-015-0037-3

**Published:** 2015-12-02

**Authors:** Hiroshi Morimoto, Hideki Tanaka, Reina Ohkubo, Maki Mimura, Noriko Ooe, Akane Ichikawa, Hiroe Yukitoshi

**Affiliations:** Faculty of Psychology, Hiroshima International University, 555-36 Kurose-gakuendai, Higashi-Hiroshima, Hiroshima 739-2695 Japan; Graduate School of Psychological Sciences, Hiroshima International University, 1-5 Noboricho, Naka Ward, Hiroshima City, Hiroshima 730-0016 Japan; Department of Healthcare Equipment, Molten Corporation, 2-18-12 Kuchita-minami, Asa-kita Ward, Hiroshima City, Hiroshima 739-1794 Japan; Nursing Department, Hiroshima Prefectural Hospital, 1-5-54 Uzina-kanda, Minami Ward, Hiroshima City, Hiroshima 734-8530 Japan

**Keywords:** Self-help therapy, Sleep hygiene education, Sleep problems, Insomnia, Hospital nurses, Japanese

## Abstract

The present pilot study examined the effectiveness of self-help therapy (SHT) in hospital nurses in Japan. Twenty-five hospital nurses (22 female, mean age 39.7 ± 14.6 years) participated in a 90-min workshop covering sleep hygiene education and brief stress management. After the workshop, participants in the SHT group (*n* = 10) were asked to practice good sleep habits, relaxation techniques, and keep a sleep diary, whereas participants in the control group (*n* = 15) were not. Pre- and post-test questionnaires, including the Japanese version of the Pittsburgh Sleep Quality Index (J-PSQI), were administered at a 2-week interval to assess subjective sleep quality, sleepiness, depressive symptoms, burnout, and quality of life. Changes in outcomes by treatment conditions were compared using linear mixed models. We found significant improvement in subjective sleep quality (global PSQI scores) in the SHT group, with Cohen’s *d* of 0.40. Participants with sleep problems at pre-test in the SHT group (scoring above the PSQI-J cut-off point) showed significant reduction in sleep latency and sleep disturbance, while those in the control group did not (Cohen’s *d* of 0.48 and 0.15, respectively). No significant improvement was found in the SHT group for sleepiness, although a clinically significant change was observed for subjective sleep quality and sleepiness in this group. No significant improvement was observed in either group for depressive symptoms, burnout, and quality of life. Our results provide preliminary evidence for the effectiveness of SHT for sleep problems in hospital nurses in Japan.

## Introduction

Many hospital nurses in Japan suffer from sleep problems due to stressful work conditions, typically characterized by labor-intensive long shift work hours and a quick return to work [1]. Over half of those undertaking shift work cannot take paid holiday breaks, which have an important role in adjusting for disturbed circadian rhythm [2]. The prevalence of insomnia in female hospital nurses in Japan is three to four times higher than that in the general Japanese population [3]. Given that sleep problems can lead to errors during working time [4] and psychological impairment [5], effective sleep management treatment for hospital nurses is necessary.

Although pharmacological treatments (e.g., benzodiazepines) are effective for alleviating sleep problems [6], prolonged use of sleeping medication has shortcomings, including deterioration of daytime functioning and development of psychological dependence, tolerance, and addiction [7]. Non-pharmacological treatments, especially cognitive behavioral therapy for insomnia (CBT-I), can overcome the shortcomings of pharmacological treatments, and the efficacy of CBT-I has been confirmed by meta-analyses [8, 9]. CBT-I is recognized as a first-line treatment and maintenance therapy for insomnia [10].

Despite its advantages, CBT-I also has some drawbacks. For example, a typical course of CBT-I takes multiple sessions [9] which has a time cost for participants. Administration of CBT-I also requires trained practitioners and the initial costs involved can be high [7]. Given that hospital nurses in Japan practice in a busy setting and human resources for administering CBT-I are limited, there is a need for a brief intervention that is feasible in a busy medical setting.

Self-help therapy (SHT), a standardized psychological treatment that can be worked through independently by participants in their own homes, is an easily accessible alternative to costly treatments [7]. SHT for sleep problems includes sleep hygiene education (i.e., providing information about mechanisms of sleep, sleep problems, and sleep management) using different multimedia tools (written materials, video, and the internet), and providing support for continuing self-management of sleep through different communication tools (face-to-face, telephone, and e-mail) [7]. A recent meta-analysis showed that the effect size of SHT for insomnia and psychological impairment using cognitive behavioral techniques (e.g., sleep diary, stimulus control, relaxation, sleep restriction) is small to moderate, which is equivalent to individual treatments [7]. As SHT is less onerous, it may be useful for busy hospital nurses. However, to the best of our knowledge, there is limited evidence for the effectiveness of SHT in hospital nurses in Japan.

The present pilot study aimed to examine the effectiveness of SHT for sleep problems in hospital nurses in Japan. As SHT requires continuous sleep improvement effort by participants (e.g., a sleep diary) [7], we included a control group who were provided sleep hygiene education, but were not asked to make additional effort. We used subjective sleep quality and sleepiness as primary outcomes, and psychological variables as secondary outcomes. We chose depressive symptoms, burnout, and quality of life as secondary outcomes, as these have previously been identified as primary mental health problems for hospital nurses. Our hypotheses were:

### **Hypothesis 1**

The SHT group showed significant improvement in primary and secondary outcomes, but the control group did not.

### **Hypothesis 2**

Improvements in sleep problems are evident after SHT.

## Materials and methods

### Participants and procedures

**Fig. 1:**
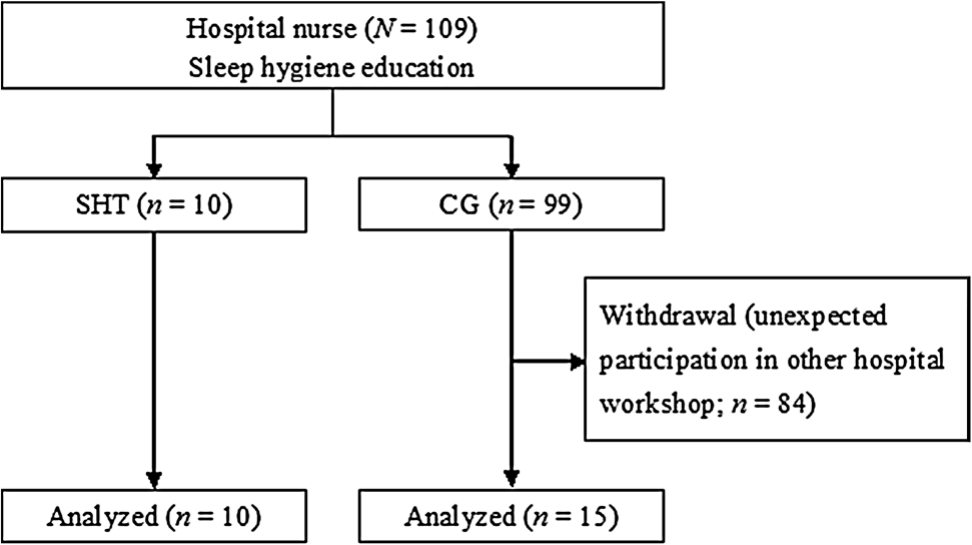
Flow of the study participants.* SHT* self-help therapy group,* CG* control group

Participants were 109 hospital nurses in a general hospital in Japan recruited through an advertisement in the hospital. Recruitment was conducted during September 2014, with assessment and intervention during October 2014. Specific inclusion and exclusion criteria were not used. Participants who agreed to keep a sleep diary for sleep improvement were allocated to the SHT group (*n* = 10), and those who did not were allocated to the control group (*n* = 99). The flow of participants is shown in Fig. 1. The present study was approved by the local ethics committee of Hiroshima International University.

Participants in both the SHT and control groups attended two 90-min sleep education workshops. In the first workshop, participants were provided sleep hygiene education. The second workshop was conducted 2 weeks after the first, and addressed how participants could apply the sleep management techniques that they learned in the first workshop to their work with patients. The series of sleep education workshops were conducted twice at different dates to be accessible to a larger number of participants. Detailed information about the second workshop has been omitted as it was not relevant to the present study. At the beginning of the first workshop, participants were given a questionnaire that included a written description about informed consent for participation in the study. At the end of the first workshop (pre-test), participants provided informed consent along with the completed questionnaire. A post-test questionnaire was conducted at the end of the second workshop, giving a 2-week interval between the pre- and post-test questionnaires.

Although 109 hospital nurses gave informed consent and provided answers to the pre-test questionnaire, 84 nurses later notified the present researchers of their withdrawal from the study because of unexpected participation in other hospital workshop that was concurrent with our second workshop. The remaining 25 participants provided answers to the post-test questionnaire.

### Measures

Sociodemographic details were collected from participants, including sex, age, working arrangements, and length of service at the hospital.

#### Primary outcomes

##### Subjective sleep quality

The Japanese version of the Pittsburgh Sleep Quality Index (PSQI-J) [11] was used to measure subjective sleep quality. The PSQI comprises 19 items and has seven components covering major aspects of sleep problems: subjective sleep quality, sleep latency, sleep duration, habitual sleep efficiency, sleep disturbance, sleeping medication use, and daytime dysfunction. Each component score ranges from 0 to 3, with a higher aggregated score of all seven components (global PSQI score) indicating greater sleep problems. Participants were asked to rate their sleeping condition in the past 1 month. We also used the PSQI-J to evaluate sleep problems. Participants who reported scores greater than the cut-off point of 5.5 were considered to have sleep problems, and those who reported scores <5.5 were considered not to have sleep problems.

##### Sleepiness

The Japanese version of the Epworth Sleepiness Scale (JESS) [12] was used to measure sleepiness. The JESS is an 8-item unidimensional scale. A higher score indicates a higher level of sleepiness, with a score of 11 or more indicating excessive daytime sleepiness. Participants were asked to rate their daytime sleepiness in the past 2 weeks. Cronbach’s alpha coefficient for the present study was 0.79.

#### Secondary outcomes

##### Depressive symptoms

The Japanese version of the Patient Health Questionnaire-9 (PHQ9) [13] was used to measure depressive symptoms. The PHQ9 is a 9-item unidimensional scale. Participants were asked to rate their experience of depressive symptoms in the past 2 weeks, using a four-point Likert scale (0 not at all, 1 several days, 2 more than half of the days, 3 nearly every day). Higher scores indicate higher depressive symptomatology. Cronbach’s alpha coefficient for the present study was 0.86.

##### Burnout

The Japanese version of the Maslach Burnout Inventory (J-MBI) [14] was used to evaluate burnout. The J-MBI consists of 17 items in three subscales: emotional exhaustion (EE), depersonalization (DP), and personal accomplishment (PA). Participants were asked to rate their burnout in the past 6 months, using a five-point Likert scale ranging from 1 (never) to 5 (daily). Higher scores on the EE and DP subscales and lower scores on the PA subscale indicate greater burnout. Cronbach’s alpha coefficients for the present study were 0.85 for EE, 0.84 for DP, and 0.66 for PA.

##### Quality of life

The Japanese version of the SF-8 Health Survey [15] was used to evaluate quality of life. This is an 8-item survey to assess health-related quality of life. Participants were asked to rate their quality of life in the past 2 weeks. The present study used two summary scores normalized in a Japanese population: physical component scores (PCS) and mental component scores (MCS). Higher PCS and MCS scores indicate higher physical and psychological functioning.

### Treatments

#### SHT

Participants in the SHT group were provided sleep hygiene education, based on previous interventions [16–18] and modified to fit a medical setting in Japan, as well as brief stress management techniques (i.e., relaxation). Subsequently, they reviewed their own sleeping habits and choose three habits from a list of good sleep habits, which they felt were possible to achieve if they worked hard. After the workshop, they were asked to practice the habits they chose and keep a sleep diary. They were also encouraged to use relaxation techniques when they felt stressed and before bedtime. Self-monitoring records about daily stress and use of relaxation techniques were integrated into the sleep diary. Further information about the sleep hygiene education used in the present study may be found in studies by Tanaka et al. [16] and Tamura and Tanaka [17, 18].

#### Control group

Participants in the control group were provided the same sleep hygiene education and brief stress management (relaxation techniques) as provided to the SHT group. Participants in the control group were not asked to practice any components of self-management.

### Data analysis

To test our hypotheses, we examined changes in primary and secondary outcomes using linear mixed models with random intercepts and restricted maximum likelihood estimation. Treatment conditions (SHT group vs. control group), measurement point (pre- and post-test), and sleep problems (present or absent), and the interaction effects of these factors were treated as fixed effects. Participants were treated as a random effect. In the analysis, the measurement point was also treated as a random effect, as the date of the pre- and post-test differed for participants who attended first or second series of workshops. Sociodemographic variables were used as control variables; sex was treated as a fixed effect, while age and length of service were treated as random effects.

We calculated two types of effect size for the primary outcomes. First, we calculated Cohen’s *d* effect size using Feingold’s formula [19]. Then, to assess clinical significance, we compared the proportion of dysfunctional individuals that moved into the functional distribution between treatment conditions, using the reliable change index (RCI) [20]. For this comparison, we used normative data presented in the original PSQI [21] for the global PSQI score, and normative data presented in the original ESS [22] for sleepiness. In both indices, individuals who reported scores greater than the cut-off points (5.5 for the PSQI-J, and 11 for the JESS) at pre-test, and those who met RCI criteria as well as falling below the cut-off points were classified as “recovered”; those who only met RCI criteria were classified as “improved”; and, those who did not met either the RCI criteria or had scores above the cutoff points were classified as “unimproved or deteriorated.”

## Results

### Participants who completed the post-test questionnaire versus those who withdrew

A comparison between participants who completed the post-test questionnaire and those who withdrew from the study without completing the post-test questionnaire is presented in Table 1. No significant differences were found, with the exception of the PA subscale of the J-MBI. Comparison of sociodemographic variables between treatment conditions showed no significant differences at pre-test: sex (female), SHT = 8, CG = 14, *χ*^*2*^ = 1.63, *p* = 0.20; mean age (years), SHT = 40.9 ± 14.8, CG = 38.9 ± 15.0, *t* = 0.32, *p* = 0.75; working arrangements (day shift and shift work), SHT = 9 and 5, CG = 7 and 3, *χ*^*2*^ = 0.09, *p* = 0.77; length of service (years), SHT = 18.9 ± 14.5, CG = 15.4 ± 14.8, *t* = 0.56, *p* = 0.58; sleep problems, SHT = 4, CG = 9, *χ*^*2*^ = 0.96, *p* = 0.33.

**Table 1:** Comparison of sociodemographic and indicator variables between participants who completed the study and those who withdrew

	Those who completed (*n* = 25)		Those who withdrew (*n* = 84)		*χ* ^*2*^	*p*	*t*	*p*
	*M*	SD	*M*	SD				
Sex (female) *n* (%)^a^	22	95.7	48	94.1	0.07	0.79		
Age	39.7	14.6	34.4	12.9			1.56	0.12
Length of service (years)	16.7	14.5	11.3	11.9			1.58	0.12
Working arrangements (shift worker) *n* (%)^b^	16	66.7	41	77.4	0.98	0.32		
A person with sleep problems, *n* (%)^c^	13	52.0	22	51.2	0.00	0.95		
Primary outcomes								
Global PSQI score	6.0	2.1	6.2	2.9			−0.31	0.76
C1: sleep quality	1.4	0.5	1.5	0.7			−0.45	0.66
C2: sleep latency	0.8	1.0	1.1	1.0			−1.41	0.16
C3: sleep duration	1.7	0.6	1.6	0.7			0.53	0.60
C4: sleep efficiency	0.1	0.3	0.2	0.4			−1.47	0.15
C5: sleep disturbance	0.8	0.4	0.7	0.5			−0.25	0.80
C6: sleeping medication	0.3	0.8	0.3	0.8			−0.15	0.88
C7: daytime dysfunction	0.9	0.9	0.8	0.8			0.58	0.56
Sleepiness	8.0	3.9	8.8	4.5			−0.78	0.44
Secondary outcomes								
Depressive symptoms	7.5	5.6	7.3	5.3			0.15	0.88
Burnout								
Emotional exhaustion	15.6	5.0	16.4	4.7			−0.65	0.52
Depersonalization	11.1	4.0	12.3	4.4			−1.23	0.22
Personal accomplishment	13.6	3.0	11.2	2.8			3.37	0.00
Quality of life								
PCS	50.6	5.6	50.9	7.7			−0.14	0.89
MCS	43.8	8.4	44.3	8.2			−0.21	0.83

*PSQI* Pittsburgh sleep quality index, *PCS* physical component scores, *MCS* mental component scores

^a^Percentages and Chi square statistic were calculated without two participants in those who completed and 33 participants in those who withdrew, as they did not report their sex

^b^Percentages and Chi square statistic were calculated without one participants in those who completed and 31 participants in those who withdrew, as they did not report their working arrangements

^c^Percentages and Chi square statistic were calculated without 41 participants who withdrew due to missing values

### Changes in primary outcomes between treatment conditions

Table 2 shows the estimated means for the primary outcomes by treatment conditions, and Table 3 shows the linear mixed models results. There were no significant differences in primary outcomes between treatment conditions at pre-test.

**Table 2:** Estimated means of primary outcomes across time by treatment condition

	All participants						Participants with sleep problems						Participants without sleep problems					
	TC^b^	Time				*d* ^a^	TC^c^	Time				*d* ^a^	TC^d^	Time				*d* ^a^
		Pre		Post				Pre		Post				Pre		Post		
		*M*	SE	*M*	SE			*M*	SE	*M*	SE			*M*	SE	*M*	SE	
Global PSQI score	SHT	6.5	2.9	5.3	3.0	0.40	SHT	8.6	4.8	6.3	4.8	0.45	SHT	4.4	2.9	4.2	3.0	1.31
	CG	5.8	3.3	6.1	3.4		CG	7.5	3.7	7.2	3.7		CG	4.0	3.8	5.1	3.9	
C1: sleep quality	SHT	1.3	2.9	1.2	2.9	1.06	SHT	1.6	4.6	1.4	4.6	0.24	SHT	1.1	2.9	1.1	2.9	2.01
	CG	1.6	3.3	1.7	3.3		CG	1.7	3.6	1.5	3.6		CG	1.5	3.9	2.0	3.9	
C2: sleep latency	SHT	1.5	1.9	1.3	1.9	0.07	SHT	2.5	3.2	1.8	3.1	0.48	SHT	0.5	1.9	0.8	1.9	−1.01
	CG	1.4	2.2	1.3	2.2		CG	2.4	2.5	2.3	2.5		CG	0.4	2.6	0.4	2.6	
C3: sleep duration	SHT	1.9	2.8	1.8	2.8	0.37	SHT	2.1	4.6	2.1	4.6	0.05	SHT	1.8	2.8	1.6	2.8	0.83
	CG	2.1	3.3	2.1	3.3		CG	2.1	3.6	2.1	3.6		CG	2.0	3.8	2.0	3.8	
C4: sleep efficiency	SHT	0.0	0.9	0.0	0.9	0.12	SHT	0.0	1.5	0.0	1.5	0.18	SHT	0.0	0.9	0.0	0.9	0.00
	CG	0.0	1.1	0.0	1.1		CG	0.1	1.1	0.1	1.1		CG	0.0	1.2	0.0	1.2	
C5: sleep disturbance	SHT	1.1	2.4	0.8	2.4	0.07	SHT	1.4	3.8	0.9	3.8	0.15	SHT	0.8	2.4	0.8	2.4	−0.23
	CG	0.9	2.8	0.8	2.8		CG	0.9	3.0	0.9	3.0		CG	0.9	3.2	0.7	3.2	
C6: sleeping medication	SHT	0.0	2.5	0.0	2.5	0.54	SHT	0.0	4.1	0.0	4.1	0.85	SHT	0.0	2.6	0.0	2.6	0.00
	CG	0.5	3.0	0.5	3.0		CG	1.1	3.2	0.9	3.2		CG	0.0	3.4	0.0	3.4	
C7: daytime dysfunction	SHT	1.1	4.4	0.7	4.4	0.15	SHT	1.9	7.1	1.1	7.1	0.74	SHT	0.2	4.4	0.2	4.4	0.43
	CG	0.4	5.1	0.6	5.1		CG	0.5	5.6	0.5	5.6		CG	0.2	5.9	0.6	5.9	
Sleepiness	SHT	8.2	8.9	7.9	8.9	0.06	SHT	8.8	14.5	7.3	14.5	0.25	SHT	7.6	8.9	8.4	8.9	−0.32
	CG	8.9	10.3	8.1	10.3		CG	8.6	11.3	8.6	11.3		CG	9.2	11.9	7.6	11.9	

*TC* treatment condition, *PSQI* Pittsburgh sleep quality index, *SHT* self-help therapy group, *CG* control group

^a^Cohen’s *d* indicates effect size of self-help therapy compared to control group

^b^SHT *n* = 10, CG *n* = 15

^c^SHT *n* = 4, CG *n* = 9

^d^SHT *n* = 6, CG *n* = 6

**Table 3:** Results of hypothesis testing by linear mixed models

	Treatment condition × measurement point		Treatment condition × measurement point × sleep problems	
	*F*	*p*	*F*	*p*
Primary outcomes				
Global PSQI score	5.09 (1,23)	0.03	0.25 (1,23)	0.62
C1: sleep quality	2.03 (1,53)	0.16	2.03 (1,53)	0.16
C2: sleep latency	1.57 (1,26)	0.22	8.46 (1,26)	0.01
C3: sleep duration	0.34 (1,16)	0.57	0.34 (1,16)	0.57
C4: sleep efficiency	0.00 (1,56)	0.99	0.00 (1,56)	0.99
C5: sleep disturbance	1.11 (1,97)	0.29	6.06 (1,97)	0.02
C6: sleeping medication	0.59 (1,30)	0.45	0.59 (1,30)	0.45
C7: daytime dysfunction	4.85 (1,107)	0.03	0.45 (1,107)	0.50
Sleepiness	0.29 (1,26)	0.59	5.49 (1,26)	0.03
Secondary outcomes				
Depressive symptoms	0.56 (1,10)	0.47	0.00 (1,10)	0.99
Burnout				
Emotional exhaustion	0.60 (1,14)	0.45	1.76 (1,14)	0.21
Depersonalization	0.08 (1,16)	0.78	3.10 (1,16)	0.10
Personal accomplishment	1.44 (1,38)	0.24	5.10 (1,38)	0.03
Quality of life				
Physical component scores	0.02 (1,26)	0.88	0.02 (1,26)	0.90
Mental component scores	0.13 (1,24)	0.72	0.28 (1,24)	0.60

*PSQI* Pittsburgh sleep quality index

**Fig. 2:**
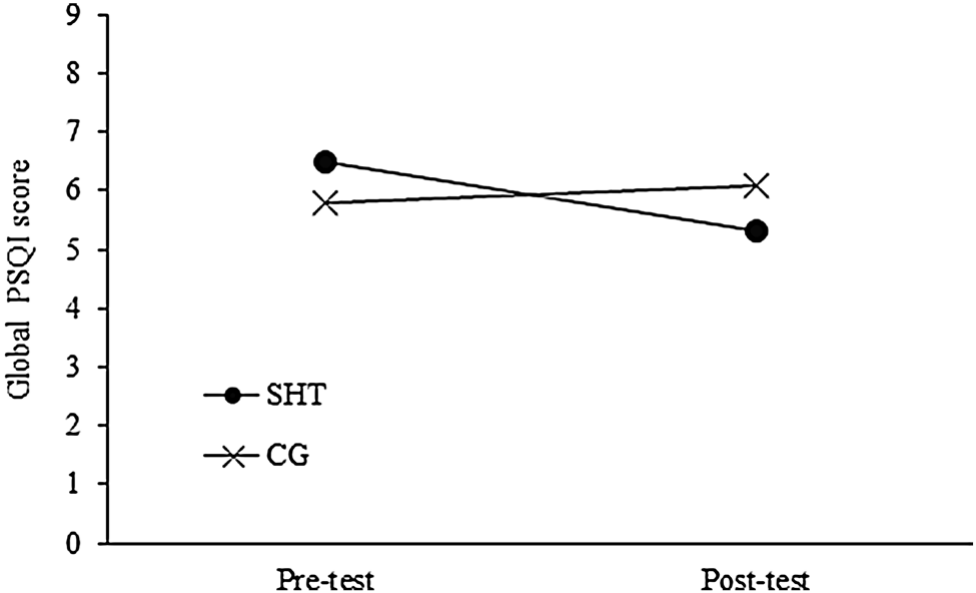
Changes in global PSQI scores across time by treatment conditions

For changes in subjective sleep quality, there was a significant interaction effect of treatment condition by measurement point for daytime dysfunction and global PSQI score. Post-hoc analysis of these interactions using the Sidak method found that for daytime dysfunction, participants in the SHT group showed a trend of reduction from pre- to post-test (*p* = 0.06), while the control group did not, with Cohen’s *d* of 0.15. Similarly, participants in the SHT group showed a significant reduction in global PSQI scores from pre- to post-test (*p* = 0.01), while the control group did not, with Cohen’s *d* of 0.40 (Fig. 2). Significant second-order interactions of treatment condition by measurement point by sleep problems were observed for sleep latency and sleep disturbance. Post-hoc analysis of these interactions using the Sidak method found that for both sleep latency and sleep disturbance, participants with sleep problems in the SHT group showed significant reduction from pre- to post-test (*ps* = 0.00), while those in the control group with sleep problems showed no reduction. Cohen’s *d* for these effects were 0.48 for sleep latency and 0.15 for sleep disturbance.

We found a significant second-order interaction for change in sleepiness. Post-hoc analysis of this interaction using the Sidak method found that participants without sleep problems in the control group showed a trend of reduction from pre- to post-test (*p* = 0.07), but this was not seen in the SHT group.

In terms of clinically significant changes in global PSQI scores, of four participants in the SHT group, two (50.0 %) were classified as recovered, one (25.0 %) was improved, and one (25.0 %) was unimproved or deteriorated. Of eight participants in the control group, one (12.5 %) was classified as recovered, one (12.5 %) was improved, and the remaining six (75.0 %) were unimproved or deteriorated. One control group participant was excluded from this calculation due to missing values at post-test. For sleepiness, three participants in the SHT group reported greater scores than the cut-off point, with two (66.7 %) classified as recovered and one (33.3 %) as unimproved or deteriorated. Four participants in the control group reported greater scores than the cut-off point, with one (25.0 %) classified as recovered and three (75 %) as unimproved or deteriorated. No participants in either the SHT or control groups were classified as improved for sleepiness.

### Changes in secondary outcomes between treatment conditions

Table 4 shows the estimated means for secondary outcomes between treatment conditions across time, and Table 3 shows the results of the linear mixed models with random intercepts and restricted maximum likelihood estimation. There were no significant differences in secondary outcomes between treatment conditions at pre-test. A significant second-order interaction effect was observed in changes in scores on the PA subscale of the J-MBI. Post-hoc analysis of this interaction using the Sidak method found that participants without sleep problems in the SHT group and those with sleep problems in the control group showed significant reduction from pre- to post-test (*ps* = 0.01).

**Table 4:** Estimated means of secondary outcomes across time by treatment condition

	All participants					Participants with sleep problems					Participants without sleep problems				
	TC^a^	Time				TC^b^	Time				TC^c^	Time			
		Pre		Post			Pre		Post			Pre		Post	
		*M*	SE	*M*	SE		*M*	SE	*M*	SE		*M*	SE	*M*	SE
Depressive symptoms	SHT	5.9	28.3	4.3	28.3	SHT	7.7	45.7	5.7	45.7	SHT	4.1	28.4	2.9	28.4
	CG	9.8	32.9	9.5	32.9	CG	9.2	35.7	8.4	35.7	CG	10.5	38.0	10.5	38.0
Emotional exhaustion	SHT	15.6	27.2	15.0	27.2	SHT	16.3	44.0	16.1	44.0	SHT	14.8	27.4	13.8	27.4
	CG	16.1	31.7	16.7	31.7	CG	18.4	34.3	17.3	34.3	CG	13.7	36.6	16.1	36.6
Depersonalization	SHT	12.6	23.2	12.7	23.2	SHT	15.3	37.5	17.0	37.5	SHT	10.0	23.3	8.4	23.3
	CG	11.8	27.0	12.3	27.0	CG	12.4	29.3	12.2	29.3	CG	11.2	31.2	12.4	31.2
Personal accomplishment	SHT	14.6	6.7	13.0	6.8	SHT	14.2	11.0	13.4	11.0	SHT	15.0	6.7	12.6	6.8
	CG	15.2	7.8	14.3	7.8	CG	16.4	8.5	15.0	8.5	CG	14.0	9.0	13.6	9.0
PCS	SHT	51.9	11.6	51.2	11.7	SHT	51.5	18.5	50.2	18.7	SHT	52.2	11.7	52.3	11.9
	CG	54.7	13.5	53.6	13.6	CG	54.1	14.7	52.0	14.9	CG	55.3	15.5	55.3	15.6
MCS	SHT	43.5	18.4	42.9	18.3	SHT	37.4	29.6	37.9	29.6	SHT	49.6	18.6	47.9	18.4
	CG	38.6	21.3	39.3	21.3	CG	37.4	23.3	37.3	23.2	CG	39.9	24.6	41.4	24.5

*TC* treatment condition, *SHT* self-help therapy group, *CG* control group, *PCS* physical component scores, *MCS* mental component scores

^a^SHT *n* = 10, CG *n* = 15

^b^SHT *n* = 4, CG *n* = 9

^c^SHT *n* = 6, CG *n* = 6

## Discussion

The purpose of the present pilot study was to examine the effectiveness of SHT for sleep problems in hospital nurses in Japan. We compared changes in subjective sleep quality and sleepiness between a SHT group and a control group as primary outcomes, and depressive symptoms, burnout, and quality of life as secondary outcomes. We also considered participant’s levels of sleep problems using the cut-off point of the J-PSQI. Overall, participants in the SHT group showed a significant reduction in global PSQI scores; those with sleep problems at pre-test showed significant reduction in sleep latency and sleep disturbance. No significant improvement was observed in either the SHT or the control groups for sleepiness and secondary outcomes. These results partially support our two hypotheses, and suggest that for hospital nurses in Japan, SHT was superior to sleep hygiene education alone (control group) for subjective sleep quality, but not for daytime sleepiness and psychological impairment.

In terms of primary outcomes, although participants in the SHT group showed a significant reduction in global PSQI scores, sleep latency, and sleep disturbance, the magnitude of these effects were small (Cohen’s *d* of 0.40, 0.48, and 0.15 respectively). However, when examining clinically significant changes in both subjective sleep quality (global PSQI score) and sleepiness, over half of the participants in the SHT group recovered, whereas few participants in the control group recovered and around 75 % were unimproved or deteriorated. Our findings suggest that although the effect sizes were small as indicated by previous meta-analysis [7], SHT may be an effective sleep management treatment for hospital nurses in Japan, and could be expected to improve sleep latency and sleep disturbance for those with sleep problems. Given that difficulty falling asleep and difficulty staying asleep are major symptoms of insomnia, our study provides preliminary evidence for the efficacy of SHT in hospital nurses.

We found no significant improvement in four of the seven PSQI component scores (sleep quality, sleep duration, habitual sleep efficiency, and sleeping medication use) and sleepiness. For habitual sleep efficiency and sleeping medication use, a floor effect was observed as the estimated means of these indicators at pre- and post-test were almost zero. The relatively higher estimated mean of global PSQI scores in our participants (both SHT and control groups) than the national average for the Japanese adult population [23] indicates that hospital nurses may have more sleep problems than the general Japanese adult population; which is consistent with the findings of Kageyama et al. [3]. However, our results also suggest that nurses have good sleep efficiency and are not likely to use sleeping medication, which is comparable with the general Japanese adult population [23].

Our finding of no significant improvement in sleep quality and sleep duration may stem from the work characteristics of hospital nurses in Japan. As many nurses engage in long shift work hours and have a quick return to work [1, 2], they may not have enough time to sleep and are likely to feel more work-related pressure. These work conditions may mean that nurses are also unlikely to experience good sleep quality regardless of improvement of sleep latency and sleep disturbance. These factors may also lead to insufficient improvement in daytime sleepiness. Another possibility suggested by our results is that the intervention duration of our study (2 weeks) was too short to allow improvement in these indices. However, this requires further investigation.

We found no significant improvement in the secondary outcomes, in contrast to the results of a previous meta-analysis [7]. Besides, participants without sleep problems in the SHT group and those with sleep problems in the control group showed a significant reduction in the PA subscale of the J-MBI. Paying attention to one’s own sleep may lead to a change in attitude toward work (e.g., is it okay to continue being devoted to work?) in particular groups of nurses. As we did not conduct a follow up survey, we do not know whether such changes in attitudes toward work increased participants’ long-term psychological adjustment. Then, this possibility also requires further investigation.

The present study has several limitations. First, participants were hospital nurses who volunteered to participate, and were not representative of hospital nurses in Japan. However, their subjective sleep quality profiles as assessed by the PSQI were generally in line with those reported by previous studies [1, 2]. To generalize the results of our study, further investigation that includes participants with high levels of insomnia and psychological impairment will be necessary. Second, because many nurses in the control group withdrew due to unexpected participation in another hospital workshop, the small sample size of this study decreased the statistical power for detection of our hypothesized interaction effects. For example, depressive symptoms decreased from pre- to post-test in the SHT group and this decrease was significant when a paired *t* test was used [*t* (8) = 2.33, *p* = 0.05]. The linear mixed model showing no significant decrease may have resulted from greater standard error for depressive symptoms stemming from the small sample size. Individuals who showed reduction in global PSQI score improved emotional exhaustion (estimated mean = 15.1, SE = 13.6 at pre-test; estimated mean = 13.2, SE = 13.6 at post-test), but those who did not, showed no improvement (estimated mean = 14.1, SE = 9.0 at pre-test; estimated mean = 14.5, SE = 13.6 at post-test) [*F* (1, 40) = 5.42, *p* = 0.03]. Higher proportion of individuals who were classified as recovered in the SHT group than in the control group suggests potential efficacy of SHT for psychological impairment. The small sample size also prohibited us from examining the potential buffering effect of working arrangements on the effectiveness of SHT.

Third, we did not collect data on daily habits (e.g., smoking status and drinking alcohol), which are thought to impact on sleep problems. Participants’ daily habits may therefore have confounded our results. Fourth, the weak research design of the present study gives rise to concern about selection bias. Given that participants in the SHT group were those who agreed to make a sleep management effort, there is a possibility that expectancy bias affected our results. As an essential element of the SHT was continuous sleep improvement effort by the participants themselves [7], we provided sleep hygiene education to the control group to clarify the effectiveness of SHT. Therefore, some participants in the control group might have voluntarily made an effort to improve their sleep based on the sleep hygiene information provided, which might explain the equivocal results of our study. Finally, as noted above, we did not conduct a follow up survey, meaning that long-term effects of SHT remain unclear. Therefore, further investigation using rigorous methods (e.g., a randomized controlled trial) is necessary.

Despite these limitations, our study provides preliminary evidence for the effectiveness of SHT for sleep problems in hospital nurses in Japan. Although the efficacy of CBT-I has previously been confirmed, application of CBT-I with busy hospital nurses may not be feasible due to the load required for both practitioners and participants. Our results showed that SHT delivered via brief workshop with take-home techniques may be effective for ameliorating subjective sleep quality in hospital nurses, and provides preliminary support for the feasibility of SHT with nurses in a busy hospital setting. Meanwhile, our results also showed that SHT was less effective for some aspects of sleepiness, depressive symptoms, burnout, and quality of life, which may stem from the characteristics of individual nurses and their attitudes toward their work conditions. This indicates that improvement of the SHT that takes these factors into account will be needed.
